# Categories of variables in analysis of genetic diversity in S_1_ progenies of *Psidium guajava*

**DOI:** 10.1038/s41598-022-26950-0

**Published:** 2022-12-26

**Authors:** Moisés Ambrósio, Alexandre Pio Viana, Derivaldo Pureza da Cruz, Sandra da Costa Preisigke, Natan Ramos Cavalcante, Deurimar Herênio Gonçalves Júnior, Bruno Dias Amaral, Antônio Teixeira do Amaral Junior, Josefa Grasiela Silva Santana, Jocarla Ambrosim Crevelari, Rogério Figueiredo Daher, Yure Pequeno de Souza

**Affiliations:** 1 Universidade Estadual Do Norte Fluminense Darcy Ribeiro (UENF), Av. Alberto Lamego, 2000, Parque Califórnia, Campos dos Goytacazes, Rio de Janeiro zip code: 28013-600 Brazil; 2 Universidade Federal de Viçosa (UFV), Av. Peter Henry Rolfs, S/N - C, Viçosa, Minas Gerais Brazil

**Keywords:** Genetics, Plant sciences

## Abstract

Crossing and developing inbred lines have been promising options for guava breeding programs. The purpose of this study was to evaluate the genetic divergence among genotypes of S_1_ inbred guava families by means of the Gower’s technique and the Ward-MLM methodology, to verify the correlation and relative contribution of traits, as well as to identify descriptors with minimum efficiency for this species. The experiment was implemented at the Estação Experimental da Ilha Barra do Pomba, in the municipality of Itaocara, RJ, Brazil. A randomized block design with 18 inbred families, three replicates, and ten plants per plot was used for the experimental design. After 19 months from the planting of the experiment, the 61 earliest and most productive genotypes (individual plants) were evaluated. For this purpose, 29 descriptors were evaluated, of which fifteen were qualitative and fourteen, quantitative. The characteristics required to obtain the distance matrix were analyzed based on the Gower algorithm, and a comparative cluster between the dendrograms of the morphoagronomic variables was achieved from this matrix. Lastly, the Ward-MLM procedure was applied to form the clusters of inbred families. By using all 29 descriptors, greater efficiency was achieved in cluster discrimination. Hence, according to the results identified, it is not possible to indicate minimum descriptors for the culture. Using the Ward-MLM method, the descriptors that most contributed to the divergence among the genotypes were fruit flesh mass, fruit weight, fruit diameter, fruit flesh thickness, fruit placental mass, and fruit length. The most divergent genotypes can be recommended for further crosses or self-pollinations to develop new lines in the guava breeding program of UENF.

## Introduction

Guava has a special position among tropical fruits in Brazil, mainly due to the great variety of products and by-products, uses, and forms in which it is consumed. In the state of Rio de Janeiro, guava is a suitable fruit in view of the edaphoclimatic conditions, favourable soils, and proximity to the port facilities, thereby benefiting the commercial production of guava trees. As such, the *Universidade Estadual do Norte Fluminense Darcy Ribeiro* (UENF) has been conducting an breeding program for guava trees for approximately ten years, with positive results so far in the selection and management of new genotypes^1–5^^.^

Under this scenario, guava tree breeding with a focus on the selection of elite genotypes via segregating populations has demonstrated to be an efficient selection strategy. It was, however, necessary to evaluate partial inbreeding on the various traits of importance for the guava tree breeding, mainly in the maintenance and fixation of the desired agronomic characters in the genotypes evaluate^6^.

According to Ambrósio et al.^6^ using S_1_ inbred families, in which plants with high yield and precocity could be observed, in addition to high homogeneity within the families, was shown to be effective. On the basis of what was stated above, it became necessary to conduct studies to obtain lines, to fix favorable alleles in genotypes of interest, and also to obtain hybrids, making available to producers a homogeneous and of high-quality material for orchards in various growing regions of Brazil.

In this assumption, one of the aspects of utmost importance for the plant breeding to select superior cultivars is the genetic divergence study, because it allows knowing the magnitude of the genetic variability of populations, enabling the monitoring of crosses, self-fertilization, and acquisition of viable information for the preservation and use of plant genetic resources^7^. In perennial species, such as *P. guajava*, it can be carried out on the basis of morphological and agronomic descriptors, characteristics directly related to production and market. Among the methodologies to establish the relevance of descriptors in the population characterization, the use of principal component analysis, method of Singh, and correlation estimates are emphasized. These methodologies have been used in the study of genetic diversity in fruit trees^8,9^.

In this context, the use of multivariate techniques has made possible studies on genetic divergence among genotypes. Most of these techniques are based on algorithms, or distance measurements, which simultaneously consider numerous characteristics and allow unifying multiple information of a set of characters^10^. The Gower’s technique (1971) was proposed for simultaneous analysis of quantitative and qualitative data. As such, this combined data analysis leads to a better understanding of the aspects considered and, especially, more accurate and effective conclusions, from the statistical point of view, about the genetic relationship among the accesses studied^11^.

Another high impact technique to quantify genetic diversity among genotypes is the Ward-Modified Location Model—Ward-MLM methodology proposed by Franco et al.^12^. This method permits the simultaneous analysis of both quantitative and qualitative variables, accessing a significant part of the available germplasm information. By using this technique, the optimal number of clusters can be defined, and the real probability of each access be assigned to a given cluster can be reliably identified^13^. This procedure has been applied to some studies, such as with maize^14^; fodder turnip^15^; tomato^13^; beans^16^; pepper/sweet pepper^17^; banana^18^; and guava^2^.

In view of the considerations above, this study aimed at evaluating the genetic divergence among genotypes of S_1_ inbred guava families by means of the Gower’s technique and the Ward-MLM methodology, in addition to verifying the correlation and relative contribution of the traits, as well as identifying the descriptors with minimum efficiency for this species.

## Material and methods

### Self-pollination and obtaining the evaluated population

The 18 inbred families evaluated here came from populations developed by Pessanha et al.^1^ in a pre-breeding study. After molecular analysis, intraspecific crosses of *Psidium guajava* accesses were performed. These crosses were performed among the seven superior and contrasting parents, found via RAPD molecular analysis, resulting in 17 segregating families. This segregating population of wide genetic variability was subsequently evaluated and selected by Quintal et al.^5^ via REML/BLUP, in which the most productive progenies were selected and self-fertilized to originate the 18 inbred families that comprise this experiment (Table 1).

**Table 1:** Origin and development of S1 families of guava. UENF, Campos dos Goytacazes, RJ, 2022.

Full-sibling families *Parents of S_1_ plants/families	Developed S_1_ families	Full-sibling families *Parents of S_1_ plants/families	Developed S_1_ families
F17/G5/B1	Family 1	F10/G5/B1	Family 10
F7/G9/B1	Family 2	F5/G4/B1	Family 11
F13/G3/B1	Family 3	F2/G6/B2	Family 12
F4/G6/B1	Family 4	F8/G4/B1	Family 13
F5/G8/B1	Family 5	F5/G9/B1	Family 14
F4/G5/B2	Family 6	F3/G11/B1	Family 15
F13/G4/B1	Family 7	F3/G5/B1	Family 16
F5/G10/B1	Family 8	F4/G9/B1	Family 17
F3/G7/B1	Family 9	F4/G8/P1	Familia 18

*Parents of S_1_ plants/family: Family/Genotype/Block: most productive genotypes evaluated and selected by Quintal et al.^5^ that were inbred and gave rise to S_1_ plants.

The study by Quintal et al.^5^ aimed to produce new varieties of guava with superior characteristics by the REML/BLUP procedure at individual level. The families selected by the authors can be highlighted by their first positions in the ranking for most of the agronomic traits and fruit quality evaluated.

Notably, guava is considered a mixed reproduction species (autogamous-allogamous)^19,20^ and has a self-pollination rate of 60%. Therefore, for being a self-compatible species^21^, inbred populations can be developed, either by self-pollination or crossing between related individuals, without inbreeding depression^22^.

Guava flowers are actinomorphic, hermaphroditic, being a potential factor for autogamy^22^. Faced with this possibility, plants were self-pollinated in the field and obtained by protecting flowers, covering buds before anthesis. The buds were identified and fruits were later protected with a paper bag. After harvesting the fruits, seeds were removed by rubbing them against a steel mesh sieve under running water. The removed seeds were left to dry at room temperature for 48 h, and constantly turned over to provide uniform drying.

Seeds from the fruits of self-fertilization were sown in tubettes (three seeds each tubette) and maintained in a greenhouse under polypropylene screens (Sombrite^®^ 50%) at the Research Support Unit (UAP), which is located in the State University of North Fluminense Darcy Ribeiro, campus of Campos dos Goytacazes-RJ (Brazil). Ambient humidity (greenhouse) was controlled by an automatic misting system activated when the ambient temperature reached 27 °C and remained in this environment for 60 days. Afterwards, the plants were transferred to the field for experiment implementation.

### Location and experimental design

The experiment was conducted at the experimental station of *Ilha Barra do Pomba* (Barra do Pomba Island), in the municipality of Itaocara (northwest region) (21°40′ south latitude, 42°04′ west longitude, and 76-m altitude), state of Rio de Janeiro, Brazil. The soil in the area is classified as Red-Yellow Argisol^23^. An experimental randomized block design with 18 S_1_ families, three replicates, and ten plants per plot was used.

After the development of the seedlings in the greenhouse, they were planted in the field in July 2014 at a 4-m spacing between rows and 1.5-m between plants. Liming, planting and covering fertilizers were carried out according to soil analysis, in accordance with the recommendations of Costa and Costa^24^; it was also used drip irrigation.

Nineteen months after planting the 540 plants of the experiment, the earliest and most productive plants were evaluated. As a result, 61 (individual plants) of guava trees were evaluated by means of the descriptors defined for the species *P. guajava*, in line with the International Union for the Protection of New Varieties of Plants—UPOV^25^ and described in Campos et al.^2^ (Table 3).

### Phenotyping

The following morphoagronomic traits were evaluated: (1) Stem—Stem diameter at 10 cm from the soil; branch height; and color of the stem in young buds in the 61 genotypes, as well as five young and fully developed leaves and five fruits per plant; (2) Leaves—Qualitative descriptors in the young leaves were the presence of anthocyanin and its intensity in the staining. In the fully developed leaves, the shape; curvature in the cross section and central vein; green color; base and tip shape were evaluated. Length; width; length/width ratio; and secondary leaf vein spacing were also examined; (3) Fruits—Final stalk shape; skin color; surface texture; calyx cavity diameter in relation to the one of the fruits; flesh color; and external flesh thickness in relation to the core diameter were all evaluated. Quantitative descriptors for fruit were number; fruit weight; fruit placental mass; fruit flesh mass; length; diameter; shape index; and flesh and placental thickness of the fruit.

### Analysis of genetic diversity by the Gower’s technique and clustering by the UPGMA method

In the analysis of genetic diversity, multivariate methods were adopted, applying quantitative and qualitative variables. Firstly, the Gower index^26^ was used to evaluate the quantitative and qualitative traits. Matrices and later dendrograms were constructed. At first, the main matrix, which contains the 29 descriptors, was constructed and served as a reference for data analysis. Subsequently, the matrices comprising the descriptors of stem; leaves; and fruits were constructed separately. An estimate of the dissimilarity index, ranging from 0 to 1, was generated.

The dissimilarity was obtained by

1 $$ Sij = \frac{{\sum\limits_{k = 1}^{p} {W_{ijk} } \cdot S_{ijk} }}{{\sum\limits_{K = 1}^{p} {W_{ijk} } }}, $$

in which:i and j = individuals to be compared regarding characteristic k;p = total number of characteristics; and*S*_ij_ = contribution of variable k to the total distance.

If the variable was qualitative, ***S***_**ijk**_ considered value **1** when there was positive or negative agreement for characteristic **k** between individuals **i** and **j**; otherwise, when the variable is quantitative, there is

2 $$ S_{ij} = \frac{{\left| {Y_{ik} - Y_{jk} } \right|}}{{R_{k} }}, $$

in which:R_k_ = the amplitude of variation of variable k, assuming values **0** and **1** or integers between them.

The **w**_**ijk**_ value was a weight applied to define individual ***S***_**ijk’s**_ contributions. Under this aspect, when the value of variable k was absent in one or both individuals, **w**_**ijk**_** = 0**; or, if not, it was **1**.

On the basis of the distance matrices generated, individuals were clustered by the Unweighted Pair Group Method with Arithmetic Mean—UPGMA, and the distance matrices with the 29 variables were compared with the distance matrices with fewer variables (leaves and fruits) using the Dendextend package in the R program (http://www.r-project.org).

### Relative importance of traits

As a suggestion for discarding the less informative quantitative descriptors, it was applied a method based on the relative importance of the traits^27^, in which this relative importance was estimated by means of the participation of the components, concerning each of the traits, in the total dissimilarity observed. The analysis was conducted by using the Genes computer program^7^.

### Pearson correlation

Pearson correlation was employed to verify the correlation among the quantitative descriptors, considering that the traits that can be discarded must be correlated to others selected. The correlation coefficient significance was examined by the t test. Statistical analyses were carried out with the help of the R Program^28^.

### Analysis of genetic diversity by the ward-MLM method

After the analysis carried out by the Gower Index, the Ward-MLM (Ward-Modified Location Model) method proposed by Franco et al.^12^ was used, as reported by Viana and Resende^29^. Next, it was established the ideal number of clusters following the criteria of pseudo–F and pseudo T^2^ using the Ward clustering method^30^. Considering the optimal number of clusters, the hierarchical classification was obtained by the Ward method, which gives the parameters required to implement the final step of the MLM model^31^.

Differences among the clusters, the correlation between the variables and canonical variable (CV) were graphically analyzed. These analyses were made using the SAS statistical software^32^. Diagrams were obtained using the Sigma Plot software, version 11.0.

### Ethical approval and informed consent

The research does not need ethical standards, because it has no Human and/or Animal participants. The article meets ethical standards. The research does not involve human participants and animal welfare.

## Results and discussions

### Comparative analysis and clustering using the UPGMA method

**Figure 1:**
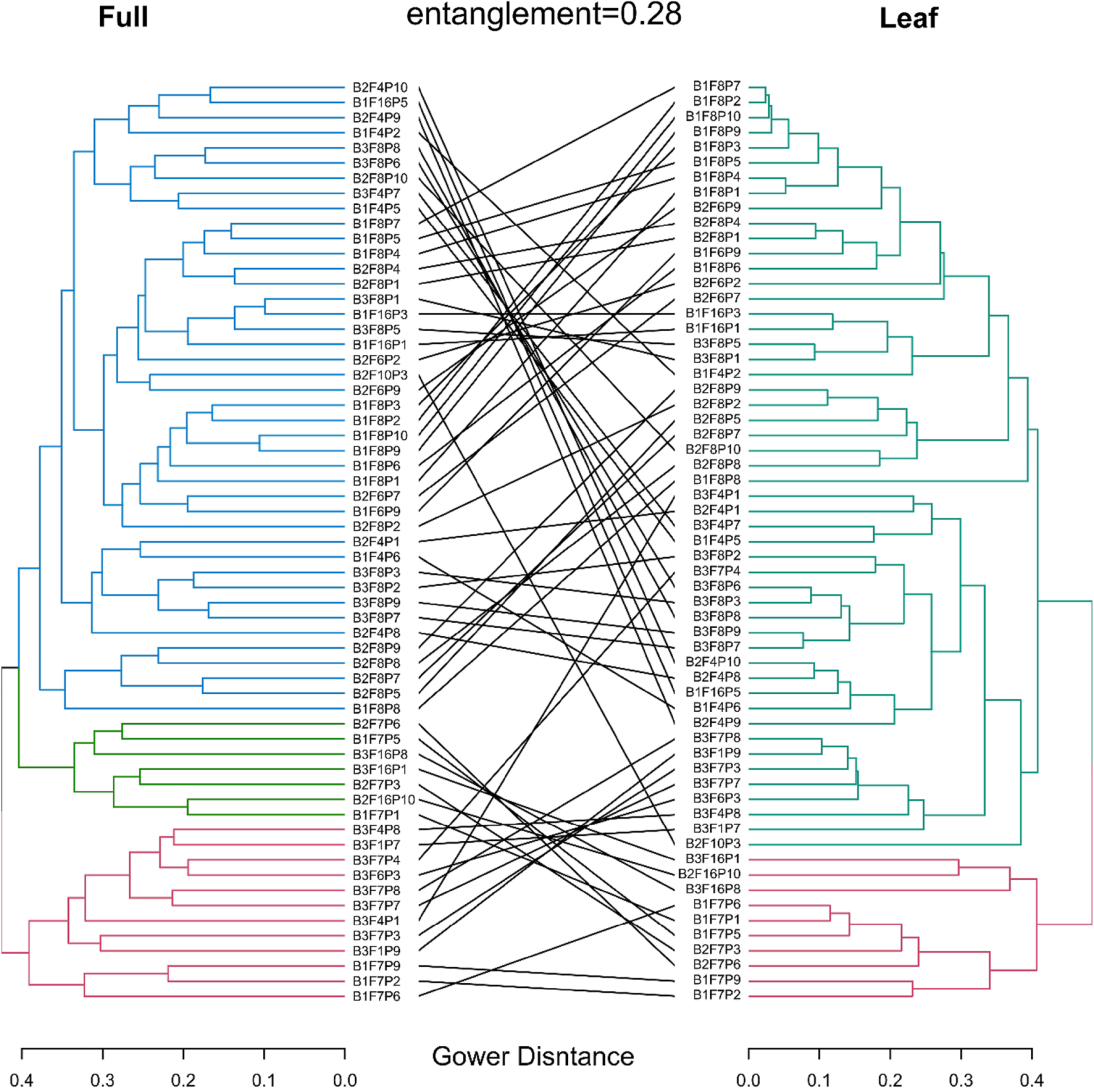
Entanglement among 61 genotypes of S_1_ inbred families of *Psidium guajava*, obtained by the Dendextend package, based on Gower Distance considering the morphoagronomic variables of fruit; leaf and stem; and only of leaf/stem. UENF, municipality of Campos dos Goytacazes, 2022.

From the dissimilarity matrix generated by the quantitative and qualitative variables, it could be established the discrimination among the genotypes of the S_1_ inbred families of *Psidium guajava*. In the clustering analysis of the 61 genotypes, the UPGMA method enabled the formation of three distinct clusters in which all the 29 morphoagronomic descriptors were considered, indicating genetic diversity among the genotypes under evaluation. Cluster I comprised 42 genotypes; cluster II, 7 genotypes; and cluster III, 12 (Fig. 1).

The purpose of this clustering analysis is to gather genotypes into clusters so that there is homogeneity within the cluster and heterogeneity among them. Among the multivariate analysis methods to quantify genetic diversity, the UPGMA is highlighted for being a technique that uses unweighted means of dissimilarity measurements, which avoids characterizing dissimilarity by extreme values (minimum and maximum) among the genotypes considered^7^.

In the comparative analysis between dendrograms with all descriptors, and dendrograms with only leaf/stem and fruit descriptors, the need for different descriptors to characterize the genetic divergence of *Psidium guajava* is evident, since both the number of clusters and the arrangement among genotypes were not kept equal in the analyses. The entanglement value, which measures the matching of genotypes between distinct dendrograms, ranges from 0 to 1, in which 0 means fully matching dendrograms and 1, dendrograms without any matching. In this way, in the comparison of the dendrograms, it was seen the greatest entanglement was 0.28 in the dendrogram showing the leaf/stem descriptors, demonstrating divergence in the distribution of the genotypes in the dendrograms (Fig. 1).

**Figure 2:**
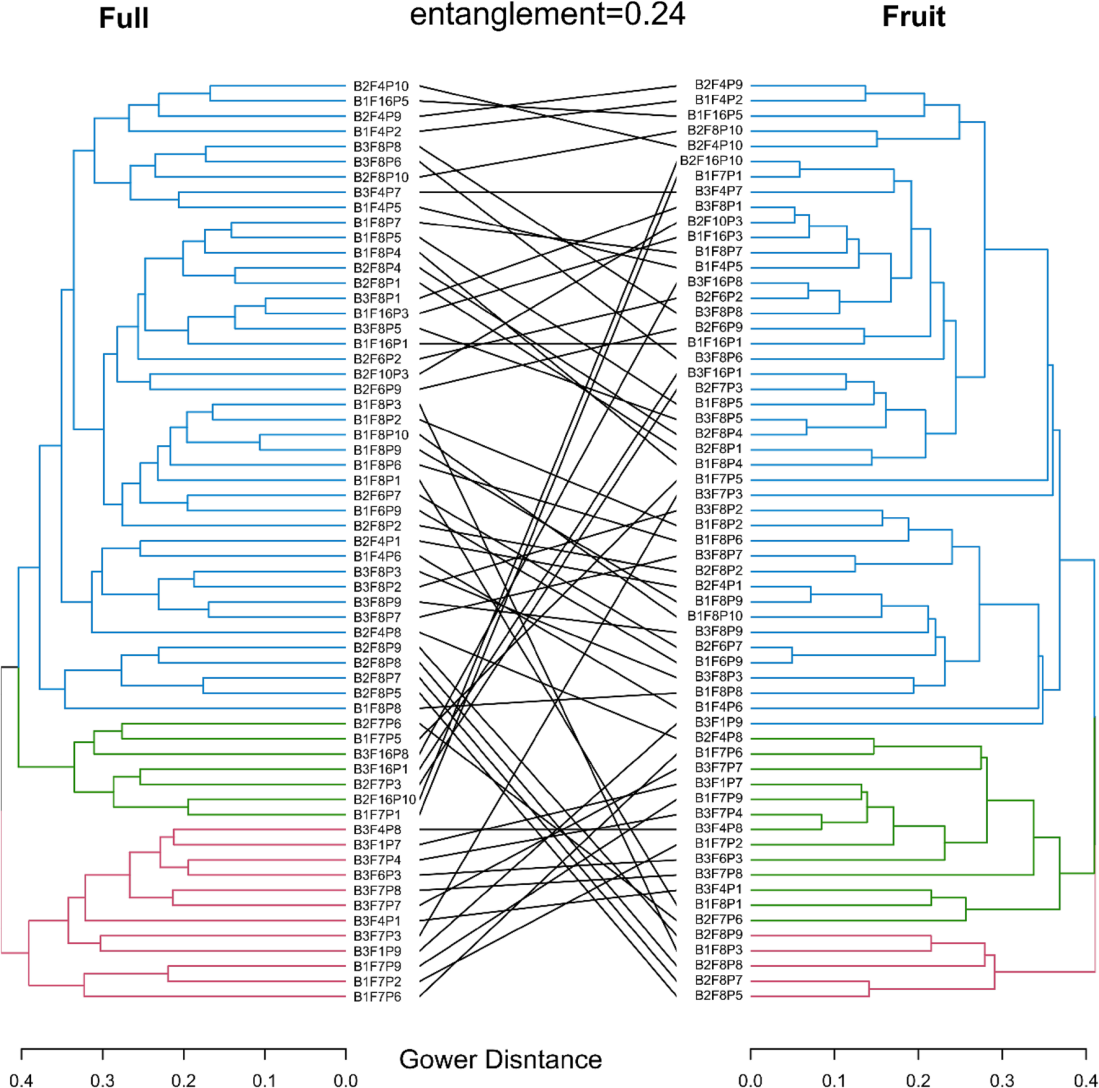
Entanglement among 61 genotypes of S_1_ inbred families of *Psidium guajava*, obtained by the Dendextend package, according to Gower Distance considering the morphoagronomic variables of fruit; leaf and stem; and only of fruit. UENF, municipality of Campos dos Goytacazes, 2022.

Clusters I and II were formed by 51 and 10 genotypes, respectively. When compared with the dendrogram containing only fruit descriptors, it presented entanglement of 0.24 (Fig. 2). The formation of three clusters was also identified, with cluster I consisting of 43 individuals; cluster II, of 13; and cluster III, of 5 genotypes.

In view of the results achieved by the comparative analysis between the dendrogram that includes all 29 descriptors with the dendrogram that only contains leaf/stem or fruit descriptors, it is necessary to use different descriptors to characterize the genetic divergence among the S_1_ inbred families of *Psidium guajava*.

Therefore, there is no possibility of indicating minimum descriptors for the culture given the results found. Similarly, by using the Dendextend package when comparing the dendrograms, with all the descriptors and the dendrogram containing only flower, leaf, or fruit descriptors, Santos et al.^33^ made it clear that different descriptors are required to characterize genetic diversity in Passiflora, because both the number of clusters and the arrangement between genotypes did not remain the same.

### Relative importance of traits

Table 2 depicts the relative contribution of the traits evaluated for diversity (Sj) and their percentage values, which constitute the measure of the relative importance of variable j for the study of genetic diversity. Following the Singh method^27^, adopted to evaluate the relative contribution of the 14 quantitative traits, it was determined that three of these traits contributed to the genetic divergence with 98.80%, while the others contributed with only 1.20%. The traits with the highest relative contribution values and which contributed most to the genotype differentiation were fruit weight (91.95%); placental weight (3.68%); and fruit flesh mass (3.15%). As such, under this criterion, it can be confirmed that these descriptors are important in the characterization of the guava genotypes evaluated, since they contribute significantly (more than 1.0% of the total variation) to the divergence discrimination.

**Table 2:** Relative contribution of 14 quantitative morphoagronomic descriptors of *Psidium guajava* for genetic divergence by the Singh method. UENF, municipality of Campos dos Goytacazes, 2022.

Variable	Sj	Value in %	Variable	Sj	Value in %
FN	2,812,934.0	0.94	FFT	18,968.473	0.00
FW	274,565,889.88	91.95	FPT	92,401.496	0.03
FFM	9,422,903.2574	3.15	SD	280,333.42	0.09
FPM	11,009,066.3	3.68	LL	3435.48	0.00
FL	214,773.6568	0.07	LW	1723.7072	0.00
FD	162,477.8932	0.05	LF/FW	137.4554	0.0
FSI	23.7092	0.0	SLVS	92.3556	0.0

*FN* fruit number, *FW* fruit weight, *FFM* fruit flesh mass, *FPM* fruit placental mass, *FL* fruit length, *FD* fruit diameter, *FSI* fruit shape index, *FFT* fruit flesh thickness, *FPT* fruit placental thickness, *SD* stem diameter at 10 cm from the soil, *LF* leaf length, *LW* leaf width, *LF/WR* leaf length/width ratio, *SLVS* secondary leaf vein spacing.

As the fruit shape; leaf length/width ratio; and secondary leaf vein spacing traits did not provide relative contribution, they can be recommended for disposal. The other variables were poorly informative to evaluate the genetic dissimilarity of guava trees, considering that they had estimates of relative contribution of small magnitudes. In the case of some descriptors, however, low variability can be explained by the first generation of self-fertilization made, which provides an increase in homozygosity and a decrease in heterozygosity in the progeny, allowing, in this manner, the achievement of more homogeneous genotypes and a consequent allele fixation^34^.

### Pearson correlation

**Figure 3:**
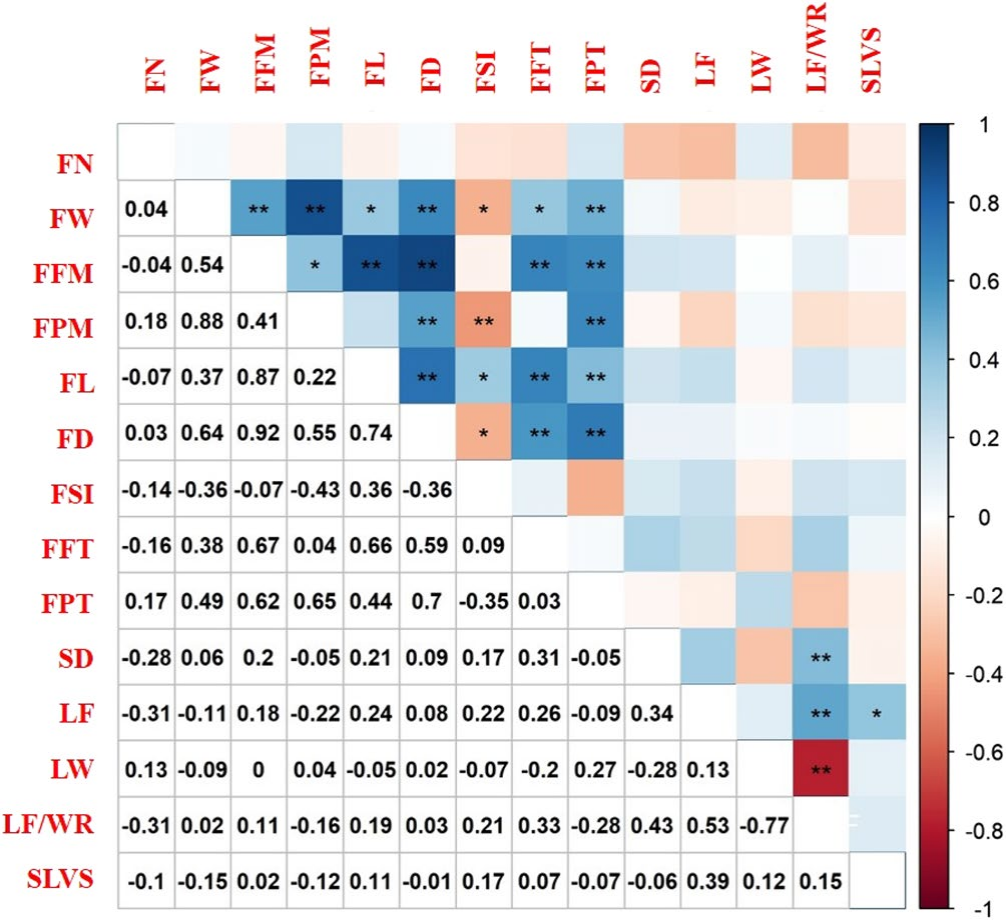
Linear correlation matrix (Pearson) among *Psidium guajava* traits obtained from 61 genotypes. UENF, municipality of Campos dos Goytacazes, 2022. Fruit number (FN); Fruit weight (FW); Fruit flesh mass (FFM); Fruit placental mass (FPM); Fruit length (FL); Fruit diameter (FD); Fruit shape index (FSI); Fruit flesh thickness (FFT); Fruit placental thickness (FPT); Stem diameter at 10 cm from the soil (SD); Leaf length (LF); Leaf width (LW); Leaf length/width ratio (LF/WR); Secondary leaf vein spacing (SLVS).

Estimates of phenotypic correlation values, resulting from the evaluation of 61 guava genotypes, are depicted in Fig. 3. To explain the relationships between the traits of economic importance, correlation estimates should be considered satisfactory, that is, r >  ± 0.50^35^. The fruit weight was highly correlated with fruit flesh mass (0.54); placental weight (0.88); and fruit diameter (0.64). The fruit flesh mass was highly correlated with fruit length (0.87); fruit diameter (0.92); flesh thickness (0.67); and placental thickness (0.62).

These correlations with fruit weight were expected, considering that genotypes with heavier fruits typically produce wider fruits with, consequently, larger flesh mass. Hence, these correlation estimates allow forecasting the response of a trait when performing the selection in another correlated one, in other words, it enables, for example, the selection in a trait of easy measurement to obtain gains in another of difficult measurement^36^.

### Clustering analysis by means of the Ward-MLM strategy

**Figure 4:**
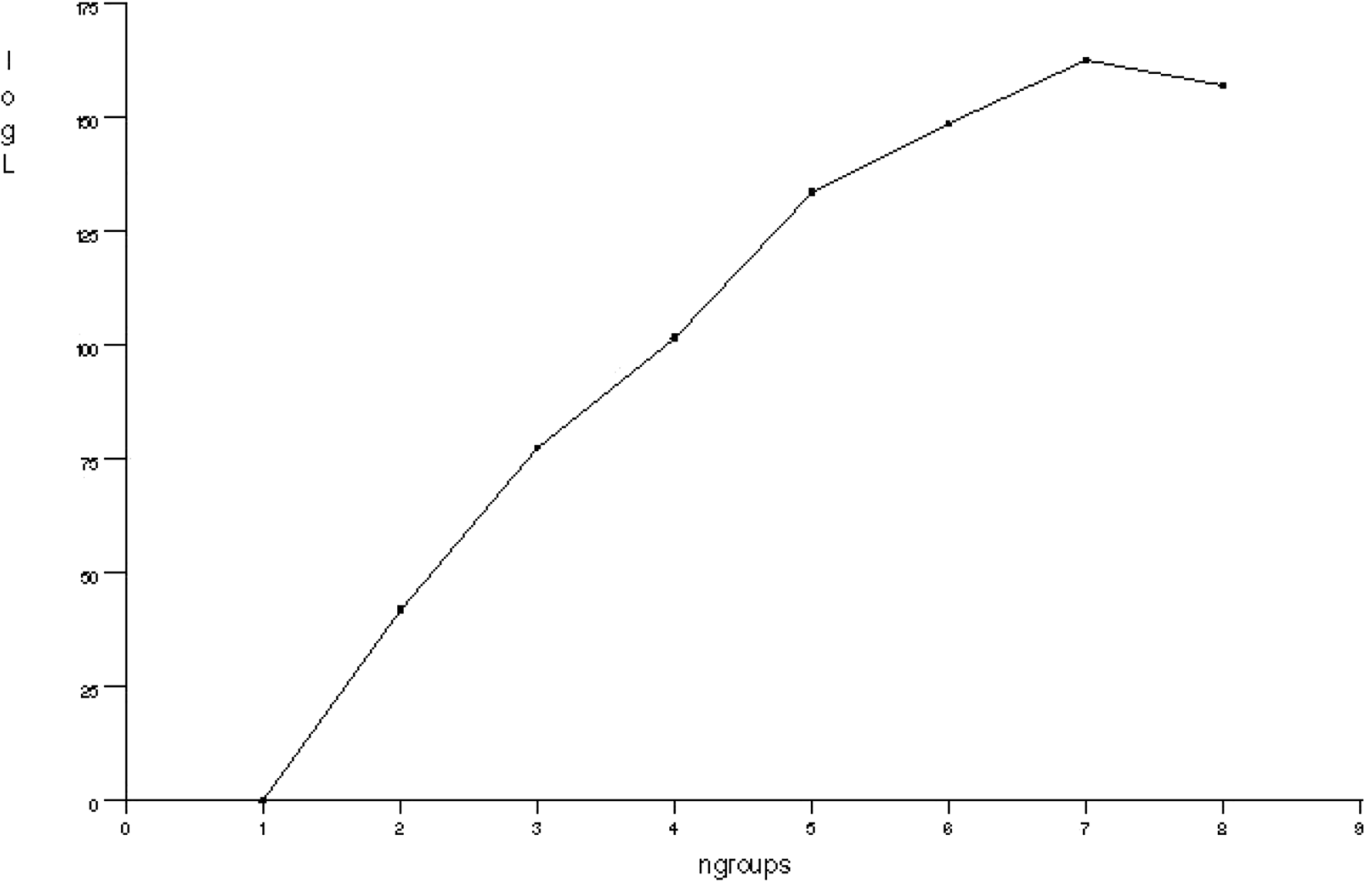
Log-Likelihood function, indicating the number of clusters formed by the Ward-MLM strategy. UENF, municipality of Campos dos Goytacazes, 2022.

In accordance with the Ward-MLM strategy, two clusters were formed following the criteria of pseudo-F and pseudo t^2^ (Fig. 4). The ideal number of clusters where there was the greatest increase in logarithmic function was verified, and the highest absolute value of 41.98 was confirmed. The indication of the number of clusters is an innovative aspect of the Ward-MLM procedure, in comparison with other hierarchical methods, which results in a more precise and less subjective cluster formation^13,37^.

The study conducted by Campos et al.^2^ with 138 guava genotypes obtained from biparental controlled crossings determined 8 the ideal number of clusters, with an increment value of 67.51. The authors noted a considerable degree of heterozygosity and wide genetic variability in these genotypes for being a segregating population. As such, it is worth mentioning that the results are different from the ones in this study, in which only the formation of two clusters was verified. Such fact seen among the genotypes of the S_1_ families is due to the reduction of genetic variability within the families and, consequently, to the increase in the frequencies of homozygosity in the first generation of self-fertilization.

Clusters I and II were formed by 33 and 28 genotypes, respectively. Genotypes were classified into the 49 phenotypic classes of the 15 qualitative descriptors evaluated, demonstrating a low genetic variability among the genotypes of the S_1_ inbred families of guava trees studied (Table 3). As for the qualitative traits, the descriptor about the branch altitude was (6.5%) erect and (65.5%) pending. The color trait of the stem was (39.2%) green; (42.6%) yellow green; and (18.1%) dark red.

**Table 3:** Phenotypic classes of plant descriptors; leaf and fruit; and number of genotypes of S_1_ inbred guava families per cluster formed by the Ward-MLM strategy.

Descriptors	Classification	Clusters		Total (%)
		I (33)	II (28)	
Tree: branch height	Erect	2	2	6.5
	Pending	31	26	93.5
Young bud: stem color	Green	12	12	39.3
	Green yellow	14	12	42.6
	Dark red	7	4	18.1
Young leaf: anthocyanin	Absent	5	5	16.4
	Present	28	23	83.6
Young leaf: intensity of the anthocyanin staining	Weak	21	11	52.5
	Medium	12	17	47.5
Fully developed leaf: shape	Ovate	2	3	8.2
	Obovate	20	15	57.4
	Obovolanceolate	2	2	6.6
	Oblong	9	8	27.8
Fully developed leaf: curvature in the cross section	Medium	13	8	34.4
	Strong	20	20	65.6
Fully developed leaf: curvature of the central vein	Absent	24	12	59.0
	Present	9	16	41.0
Fully developed leaf: green color	Green yellow	9	5	23.0
	Gray green	15	14	47.5
	Green	8	8	26.2
	Dark green	1	1	3.3
Fully developed leaf: base shape	Obtuse	4	3	11.5
	Rounded	20	19	63.9
	Heart-shaped	9	6	24.6
Fully developed leaf: tip shape	Apiculate	–	1	1.6
	Obtuse	5	4	14.8
	Rounded	28	23	83.6
Fruit: final stalk shape	Broadly rounded	8	3	18.1
	Rounded	10	6	26.2
	Truncated	15	18	54.1
	Neck-shaped	–	1	1.6
Fruit: skin color	Pale green yellow	10	4	23.0
	Pale yellow	18	18	59.0
	Dark yellow	5	4	14.7
	Orange	–	2	3.3
Fruit: surface texture	Soft	29	28	93.4
	Rough	4	–	6.6
Fruit: calyx cavity diameter in relation to that of the fruit	Small	3	9	19.7
	Medium	24	15	63.9
	Big	6	4	16.4
Fruit: flesh color	Cream	2	8	16.4
	Pale pink	11	3	23.0
	Pink	8	5	21.3
	Dark pink	11	12	37.7
	Orange pink	1	–	1.6
Fruit: external flesh thickness in relation to the core diameter	Very thin	2	–	3.3
	Thin	4	–	6.6
	Medium	23	19	68.8
	Thick	4	9	21.3

UENF, municipality of Campos dos Goytacazes, 2022.

There is, in the descriptors, a strong differentiation in the magnitude of the anthocyanin presence in leaves, with percentages of 16.4% and 83.6%, respectively. With respect to the intensity of anthocyanin staining in leaves, it can be seen that, in 52.5%, there was a weak shade, while in 47.5%, it was medium. In fully developed leaves, most genotypes had an obovate shape (57.4%), with the base shape (63.9%) and the tip (83.6%) rounded. In addition, 59% of genotypes had no curvature of the central vein in the leaves. Among those who did, 65.6% were classified as strong central vein curvature.

For the predominant leaf color, green gray was found in 47.5% of the genotypes, followed by green (26.2%); yellow green (23%); and dark green (3.3%).

In relation to fruit descriptors, the genotypes presented final truncated stalk shape (54.1%); rounded (26.2%); broadly rounded (18.1%); and neck-shaped (1.6%). In terms of skin texture, 93.4% of the genotypes showed a soft texture and 6.6% a rough one. On the other hand, 59% of the genotypes had pale yellow skin color; 23%, pale green-yellow; 14.7%, dark yellow; and 3.3%, orange. Pereira and Nachtigal^38^ pointed out that fruits of yellowish green or yellow color, when ripe, are more commercially appreciated.

For the flesh color descriptor, genotypes were classified into five categories, with a range from cream to orange pink, but with a predominance of dark pink, which was found in 37.7% of the genotypes. Pereira and Nachtigal^38^ say that pink or reddish flesh fruits are preferred not only for the industry but also for the consumption *in natura* in the Brazilian market.

In the calyx cavity diameter regarding the fruit, 63.9% of the genotypes were classified as medium (68.8%); thick (21.3%); thin (6.6%); and very thin (3.3%). These descriptors prove to be relevant when choosing genotypes for consumption *in natura*, given that they are related to factors that depreciate the product if they are not attractive to consumers.

With respect to the quantitative descriptors, cluster II had the highest number of fruits per plant (125), followed by cluster I (111) (Table 4). In both, however, there was great variation, as seen by the minimum and maximum values of each group.

**Table 4:** Minimum, maximum, and mean values of the descriptors for each of the two groups formed by the Ward-MLM strategy in 61 genotypes of inbred S1 families of guava trees.

Descriptors	Min/max-clusters		Overall mean-clusters	
	I (33)	II (28)	I (33)	II (28)
Fruit number (unit)	31.00/111.00	32.0/125.00	57.90	71.50
Fruit weight (g)	42.8/177.20	55.2/329	60.3	253
Fruit flesh mass	35.7/140.20	45.2/242	55.5	164.11
Fruit placental mass (g)	9.5/37.00	11.2/47.9	32.5	42.4
Fruit length (mm)	61.20/85.28	65.30/101.00	74.01	77.90
Fruit diameter (mm)	51.83/76.83	58.07/82.48	64.83	70.74
Fruit shape index	0.99/1.30	1.00/1.33	1.14	1.09
Fruit flesh thickness (mm)	10.61/17.37	11.38/21.90	12.96	14.77
Fruit placental thickness (mm)	29.64/47.13	32.24/54.33	39.35	41.47
Stem diameter (mm)	51.00/67.50	45.50/66.00	49.56	53.12
Leaf length (cm)	14.16/17.22	12.50/17.38	15.56	15.21
Leaf width (cm)	6.98/9.50	6.50/9.50	8.26	7.86
Leaf length/width ratio	1.58/2.26	1.66/2.43	1.89	1.94
Secondary leaf vein spacing	0.83/1.42	0.80/1.50	1.14	1.13

UENF, Campus of Campos dos Goytacazes (Brazil), 2022.

Minimum (Min) and maximum (Max) values for each of the two groups formed.

There is still less potential for production when comparing with the Paluma cultivar, which produces an average of 188 fruits per plant. Nevertheless, as the evaluation was carried out in the first year of cultivation, this was already expected. As stated by Natale et al.^39^, guava trees bear fruits from the first year onwards and, over the course of the harvest, production gradually increases until it stabilizes. Even though the number of fruits produced by some genotypes does not come close to being found in some cultivars, the families in this study are in their first harvests with the possibility of an increase in production over the cycles.

Fruit weight trait also varied within the clusters, with cluster II having the highest value (329 g). There was high magnitudes of fruit weight, reflecting the first production, when the plants still have not expressed their full productive potential and thus produce larger fruits. In general, the trend is towards the fruit mass decreasing with the stabilization of commercial production, when the plant tends to yield a greater number of fruits. Gonzaga Neto et al.^40^ consider the increase in the average weight of the fruits to be related to the number of fruits produced by each plant; as such, the greater quantity of fruits in the plant may influence it to produce smaller fruits in weight and size because the available reserves would be used to fill a greater number of fruits, limiting the size of each one of them.

Cluster I, however, had an average fruit weight of 177.2 g. Hence, these values are in accordance with the standard recommended for consumption because, according to Lima et al.^41^ fruits ranging from 100 to 200 g can be utilized for dual purposes, in other words, they can be used both for industrial processing and for consumption *in natura*.

Cluster II also showed a higher value for the fruit flesh mass (242 g) and fruit placental mass (47.9 g), resulting in a greater fruit length (101 mm) and fruit diameter (82.48 mm). Flesh mass trait is of utmost relevance, once it contributes to the definition of the flesh yield. According to Gonzaga Neto et al.^40^, the average fruit mass is an important trait, given that, in general, fruits with the greatest flesh mass are also the largest and, in turn, they are more attractive to the consumer.

For fruit shape index, values were close among the clusters, in which the mean was of 1.14 e 1.09 for cluster 1 and 2 respectively. The analysis of these variables, separately, has little importance for the fruit characterization of guava tree genotypes^41^. On the other hand, the relationship among these variables is highly representative, since it indicates the fruit shape. Pyriform or oval fruits (LD/CD ratio greater than 1) are suitable for consumption *in natura*, and those with rounded forms (LD/CD ratio close to 1) are better indicated for industrialization^42^.

The clusters presented close means between them for placental thickness and flesh thickness of the fruit, with mean values of 39.35 and 12.96 for cluster 1, and 14.77 and 41.77 for cluster 2 respectively. For the stem diameter at 10 cm from the soil, cluster I reported a mean of 49.56 mm followed by cluster II, with 53.12 mm. For descriptors related to the leaf, clusters I and II were similar for length, width, length/width ratio, and vein spacing, which resulted in a lower mean length/width ratio of (1.89 and 1.94), presenting the characteristic of a rounder leaf. For being correlated with the leaf area of the plant, the leaf length and leaf width are significant. The leaf area is directly related to the taking advantage of solar energy, which is converted into chemical energy during the process of photosynthesis^43^.

**Figure 5:**
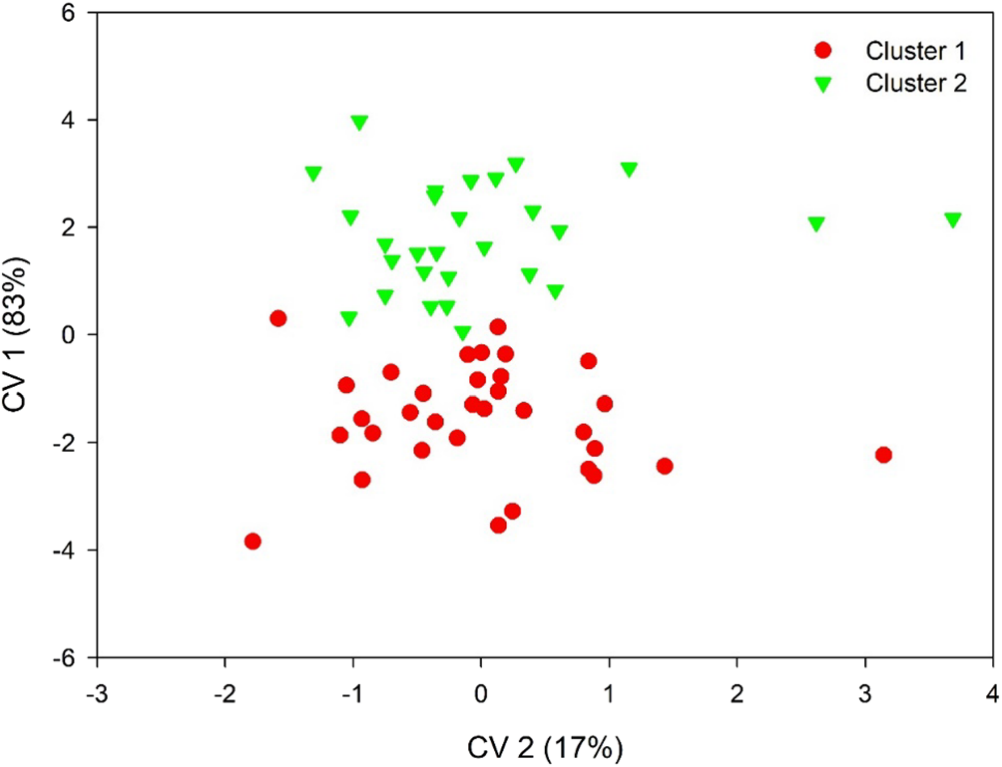
Dispersion of the first two canonical variables (CV1 and CV2) representing the formation of two clusters by the Ward-MLM strategy, based on 14 quantitative and 15 qualitative descriptors in S_1_ inbred families of guava trees. UENF, municipality of Campos dos Goytacazes, 2022.

The first two canonical variables (CV) obtained by the Ward-MLM strategy explained 100% of the total variation, being 83% of CV1 and 17% of CV2 (Fig. 5). As such, a two-dimensional representation is the most appropriate to represent the data set. The Ward-MLM procedure was applied to quantify genetic variability in studies on maize^14^; pepper/sweet pepper^15^; tomato^13^; common beans^16^; pepper/sweet pepper^17^; and banana^18^. These authors noticed the first two canonical variables explained the variability between the clusters above 80%, and the two-dimensional graph was adequate to visualize the relationship between the clusters. In this way, this high value shows the graphic representation of the first two canonical variables is adequate to verify the relationship among clusters and individuals within a cluster, enhancing the reliability of the results.

The quantitative descriptors that most contributed to quantify the genetic diversity among the genotypes, in other words, the ones that displayed the greatest correlations with the first canonical variable were fruit flesh mass (0.549); fruit weight (0.533); fruit diameter (0.515); fruit flesh thickness (0.462); fruit placental mass (0.455); and fruit length (0.295) (Table 5). In the study developed by Campos et al.^2^, with guava accesses, the authors found that the greatest correlations of the variables with the first canonical variable were flesh mass; fruit mass; fruit diameter; mean fruit weight; mesocarp thickness; and fresh placental mass.

**Table 5:** Canonical variables of 29 plant descriptors; fruit; leaf; and stem in 61 genotypes of S_1_ inbred guava families.

Descriptors	Canonical variables (CV)	
	CV1	CV2
Fruit number (unit)	0.285083	− 0.096821
Fruit weight (g)	0.533293	− 0.231880
Fruit flesh mass (g)	0.549536	− 0.200411
Fruit placental mass (g)	0.455765	0.228783
Fruit length (mm)	0.295039	− 0.147165
Fruit diameter (mm)	0.515987	− 0.239606
Fruit shape index	− 0.331385	0.142376
Fruit flesh thickness (mm)	0.462197	− 0.198141
Fruit placental thickness (mm)	0.245262	− 0.168313
Stem diameter at 10 cm from the soil (mm)	0.236233	− 0.062005
Leaf length (cm)	− 0.212856	0.045014
Leaf width (cm)	− 0.341055	0.165505
Leaf length/width ratio	0.162700	− 0.041308
Secondary leaf vein spacing (cm)	− 0.022226	0.026963

UENF, municipality of Campos dos Goytacazes, 2022.

In view of the above, it is worth emphasizing that knowing the morphoagronomic traits evaluated herein is fundamental in plant breeding programs, especially to support future crosses and/or self-fertilization of the genetic breeding program of guava trees. By characterizing the genetic diversity of the S_1_ inbred families of guava tree, the identification of genotypes agronomically superior was made possible, providing better gains as a result of their selection. This study provided a more accurate basis for choosing the best genotypes that can be selected and self-fertilized, so as to achieve the most promising genotypes within the breeding program.

## Final considerations

Comparative analysis between dendrograms revealed the use of all 29 descriptors led to greater efficiency in discriminating clusters, enabling genetic dissimilarity to be achieved more efficiently among the S_1_ inbred families of guava trees.

The Gower’s technique is an effective tool to detect genetic divergence and to cluster accesses using qualitative and quantitative variables simultaneously.

The traits with the greatest relative contribution to genetic variability in the families studied were fruit weight; placental weight; and fruit flesh mass.

The Ward-MLM statistical procedure is a useful tool in detecting genetic divergence and in clustering genotypes, and, by means of it, the descriptors that most contributed to the divergence among the guava genotypes were fruit flesh mass; fruit weight; fruit diameter; fruit flesh thickness; fruit placental mass; and fruit length.

The most promising genotypes for selection in the breeding program belong to group II, due to their good performance for all traits, especially fruit number, fruit weight, fruit pulp mass, and fruit diameter and length.

Therefore, the most divergent genotypes with high production potential can be recommended for further crosses or self-fertilization to acquire new lines in the guava breeding program at UENF.
